# Supplementary material for: Deletion of wfs1 Impairs Oligodendrocyte Precursor Cells Dorsal Distribution and Myelination Through the wfs1-hmgcs1 Axis in Zebrafish
Source: Biology (Basel). 2026 Aug 16;15(16):1402. doi: 10.3390/biology15161402 (PMC13510151; doi:10.3390/biology15161402)
Supplement: Supplementary file 1 [file biology-15-01402-s001.zip › File S1-Transcriptome sequencing data and differentially expressed genes (DEGs) in wfs1-- zebrafish at 5 dpf/2.KEGG/wfs1KOvsWT/ALL/src/dre00790.html]

dre00790


- dre:557552

- Up regulated genes

557552(1.27169280646928)

- dre:557552

- Up regulated genes

557552(1.27169280646928)

- dre:791448

- Up regulated genes

791448\_1(4.96863403887753)

- dre:557552

- Up regulated genes

557552(1.27169280646928)

- dre:100330410

- Up regulated genes

100330410(1.88154010673068)
- dre:574009

- Up regulated genes

574009(1.57400981620651)

- dre:557552

- Up regulated genes

557552(1.27169280646928)

Close
